# Involvement of medical students in a surgery congress: impact on learning motivation, decision-making for a career in surgery, and educational curriculum

**DOI:** 10.1007/s10354-020-00802-w

**Published:** 2021-01-14

**Authors:** Ibrahim Alkatout, Veronika Günther, Sandra Brügge, Johannes Ackermann, Magret Krüger, Dirk Bauerschlag, Nicolai Maass, Sebastian Lippross, Ingolf Cascorbi, Jan-Hendrik Egberts, Thomas Becker, Daniar Osmonov, Klaus-Peter Jünemann, Thilo Wedel

**Affiliations:** 1 Department of Obstetrics and Gynecology, University Hospitals Schleswig-Holstein, Campus Kiel, Arnold-Heller-Str. 3, House C, 24105 Kiel, Germany; 2 Department of Trauma Surgery and Orthopaedics, University Hospitals Schleswig-Holstein, Campus Kiel, Arnold-Heller-Str. 3, 24105 Kiel, Germany; 3 Institute of Experimental and Clinical Pharmacology, University Hospitals Schleswig-Holstein, Campus Kiel, Arnold-Heller-Str. 3, 24105 Kiel, Germany; 4 Department of General‑, Visceral‑, Thoracic‑, Transplant- and Paediatric Surgery, University Hospitals Schleswig-Holstein, Campus Kiel, Arnold-Heller-Str. 3, 24105 Kiel, Germany; 5 Department of Urology and Pediatric Urology, University Hospitals Schleswig-Holstein, Campus Kiel, Arnold-Heller-Str. 3, 24105 Kiel, Germany; 6 Center for Clinical Anatomy, Institute of Anatomy, Christian-Albrechts-Universität zu Kiel, Otto-Hahn-Platz 8, 24118 Kiel, Germany

**Keywords:** Teaching project, Students, Minimally invasive surgery, Surgical career, Curriculum, Lehrprojekt, Studenten, Minimal-invasive Chirurgie, Chirurgische Karriere, Curriculum

## Abstract

During the preclinical period of medical school, the clinical relevance of theoretical knowledge is given little attention. Medical students of the second year were invited to participate in an interdisciplinary congress for robot-assisted and digital surgery. The students had to evaluate the impact of the congress on their learning motivation, decision-making for a career in surgery, and relevance for their educational curriculum. Participation in the congress increased their learning motivation for preclinical subjects, and significantly increased their interest in a surgical career. Most students considered active involvement in medical congresses a valuable supplement to the medical curriculum. Congress participation during the preclinical period was ranked positively by medical students. Greater learning motivation and enthusiasm for the pilot teaching project as well as for surgical disciplines were registered. Thus, early involvement of medical students in scientific congresses should be an integral part of their educational curriculum.

## Abstract (de)

In den vorklinischen Semestern des Medizinstudiums wird der klinischen Relevanz oft wenig Aufmerksamkeit geschenkt. Medizinstudierende des zweiten Studienjahres wurden eingeladen, an einem interdisziplinären Kongress für robotergestützte und digitale Chirurgie teilzunehmen. Im Anschluss sollten die Studierenden den Einfluss des Kongresses auf ihre Lernmotivation, die Entscheidungsfindung für eine Karriere in einem chirurgischen Fach und die Relevanz für das Curriculum bewerten. Durch die Kongressteilnahme steigerte sich die Lernmotivation für vorklinische Fächer, und das Interesse an einer chirurgischen Laufbahn nahm erheblich zu. Die meisten Studierenden betrachteten die aktive Teilnahme an medizinischen Kongressen als wertvolle Ergänzung des Lehrplans für das Studium. Die Kongressteilnahme während der Vorklinik wurde sehr positiv bewertet. Es wurde eine größere Lernmotivation für das Medizinstudium sowie eine Begeisterung für das Pilot-Lehrprojekt und für die chirurgischen Disziplinen registriert. Daher sollte die frühzeitige Einbindung der Medizinstudenten in wissenschaftliche Kongresse ein integraler Bestandteil ihres Curriculums werden.

## Background

In most German medical schools, the curriculum is divided into a preclinical and a clinical period. The preclinical period (2 years) includes anatomy, physiology, and biochemistry as well as basic natural and social sciences such as biology, chemistry, physics, and medical sociology/psychology. The clinical period (4 years) covers all clinical subjects and familiarizes the student with the spectrum of conservative and surgical medical disciplines. The last year (sixth year) is the so-called practical year and is spent exclusively in teaching hospitals. While two trimesters have to be absolved in internal medicine and surgery, the clinical subject of the third trimester may be selected by the student.

In the USA, a student must obtain a bachelor’s degree (4 years) before being accepted at a medical school. This pre-medical education does not necessarily have to be linked to medical science. The American curriculum at medical schools is also divided into a preclinical (2 years) and clinical (2 years) study period. In Great Britain, the first year of medical school covers anatomy, biochemistry, and physiology, while the second year is focused on basic clinical subjects such as pathology, pharmacology, and neurosciences. The third year is spent in elective periods and is concluded with a Bachelor of Arts degree. The fourth and fifth years address all relevant clinical subjects and are much more practice oriented than in Germany. The sixth/final year includes the consolidation of skills and prepares the student for clinical practice [1].

The rather strict division of the traditional medical curriculum into a preclinical and clinical period, as offered in most German medical schools, has a number of didactic disadvantages. Most preclinical subjects are taught and learned without major emphasis on clinical aspects. For example, during the dissection course in macroscopic anatomy, anatomical structures are not consistently linked to relevant surgical procedures and are frequently committed to memory without being given clinical context. Subsequently, the significance of anatomical facts acquired during the preclinical period will become evident rather late during the clinical study period or even during the student’s residency [2]. Thus, there is an increasing need to bridge this gap by offering early insights into clinical applications of preclinical information. Highlighting these practical aspects is expected to enhance the students’ learning motivation as well as helping them to become identified with their future professional careers. In this context, the link between anatomical and clinical topics is particularly relevant for surgical subjects, because a thorough knowledge of anatomy is indispensable for all surgical disciplines.

However, the decision in favor of a career in surgery has undergone a marked change during the last decade. There has been a steady decline in the number of medical students opting for a surgical career. The underlying reasons for this disillusioning process are multifactorial, but social changes associated with the currently dominant “generations Y and Z” may contribute substantially to this development [3]. These generations tend to refuse what they consider outdated hierarchies and expect a meaningful work–life balance. Surgical disciplines call for substantial physical effort and time in addition to a rather long learning curve, which makes surgery less attractive [4].

Moreover, it may be assumed that the next generation of surgeons will hardly experience the traditional open surgical techniques, but will be directly confronted with minimally invasive digital surgery. Over the past decade, many surgical interventions have been converted to the laparoscopic or robotic approach [5]. While training in surgery traditionally started with open-access procedures, these were followed by laparoscopic, pelviscopic, or thoracoscopic procedures. The new generation of surgeons frequently start their professional career by performing minimally invasive surgery and are thus facing more challenging scenarios [6]. Modern formats of surgical congresses have taken these challenges into account by offering practical hands-on workshops, virtual-reality training, and tutorial video sessions or live surgeries to enhance surgical skills and education [7]. However, most congresses are confined to postgraduate education and provide limited options for medical students to be involved in their stimulating learning atmosphere.

Based on these considerations, a pilot teaching project was launched by the Medical Faculty of Kiel University. Preclinical medical students of the second academic year were invited to participate in an interdisciplinary congress on robot-assisted and digital surgery. The congress covered major surgical interventions in the fields of urology, gynecology, and surgery by means of video lectures, live surgeries, and interdisciplinary discussions. The aim of the congress was to highlight the latest technical developments in laparoscopic and robot-assisted surgery. The event was held at the end of the preclinical dissection course of macroscopic anatomy, and was actively assisted by the staff of the Institute of Anatomy. The aims of this pilot teaching project were to evaluate the impact of preclinical medical students’ participation in a surgical congress on their learning motivation and decision-making for a career in surgery, and to assess whether this concept could become an integral part of the educational curriculum.

## Methods

### Participating medical students

All medical students of the second academic year (*n* = 176) at the end of their preclinical period were officially invited to attend the congress on a voluntary basis. At the time of the convention they were in the final phase of their practical course in macroscopic anatomy, including supervised dissection of body donors and anatomy lectures. Based on the program, 2 weeks before the congress the students were asked to prepare written and illustrated excerpts of those anatomical regions which would then be presented and treated surgically during the congress. As these anatomical topics were also relevant for the upcoming written and oral examination at the end of the preclinical period, the effort was worthwhile for the students.

### Congress format

The congress was the 11th symposium of the German Society for Robot-Assisted Urology (*Deutsche Gesellschaft für Roboter-assistierte Urologie e.* *V.*, DRUS) and simultaneously the first interdisciplinary symposium for robot-assisted and digital surgery in urology, gynecology, and visceral surgery organized by the Kurt Semm Center at the University Hospital of Schleswig-Holstein in Kiel (June 5th–7th, 2019). The congress format was unconventional and innovative. Instead of a single surgical discipline, those anatomical regions (pelvis, retroperitoneum) which are of interest for all disciplines were addressed. The organizers aimed to exchange and discuss current options, developments, and potentials of minimally invasive techniques among the three major fields of surgery. Interactive lectures, video sessions, and live surgeries were presented by urologists, gynecologists, and visceral surgeons. All surgical procedures were performed on a minimally invasive basis either by the laparoscopic or robotic approach, and included the following operations: radical prostatectomy with extended lymphadenectomy, salvage lymphadenectomy after prostatectomy, colorectal resection with total mesorectal excision/complete excision of the mesocolon, total hysterectomy with para-aortal lymphadenectomy, and sacrocolpopexy.

### Pilot teaching project

The intention to make the congress accessible to preclinical students was discussed in detail and planned at the Kurt Semm Center, Kiel School of Gynecological Endoscopy, and the Institute of Anatomy. The somewhat different format of the meeting was considered especially appropriate to address the pending issues outlined in the introduction: (1) to bridge the gap between preclinical theoretical knowledge and clinical application, (2) to induce enthusiasm for major surgical disciplines, (3) to establish early contact with minimally invasive and digital surgical techniques. Based on these considerations, the Medical Faculty of Kiel University funded this pilot teaching project to enable medical students to attend the congress free of charge. All chairmen and presenters were informed about the project well in advance, so that they could consider these special circumstances in their lectures, explanatory notes, and comments during live surgeries. The students attended all sessions of a full-day program instead of regular lectures at the university. They were also permitted to visit the industrial exhibition and become familiar with the latest technical innovations and simulator devices in the field of minimally invasive and robotic surgery. They were invited to approach the surgical faculty and other participants for further questions and networking. At the end of the congress the medical students were asked to fill in a questionnaire.

### Questionnaire

The questionnaire recorded age, gender, and previous vocational training, and consisted of the following questions:

1. Did the congress increase your motivation to learn anatomy?
2. Were you able to follow the details of the individual surgical interventions?
3. How high was your interest in a professional career in surgery *before* the congress?
4. How high was your interest in a professional career in surgery *after* the congress?
5. How highly do you rate the educational gains of participating in a clinical congress as a medical student?
6. Should the participation of medical students in clinical congresses be an integral part of the medical curriculum?

The questions could be answered on a five-point Likert scale consisting of the following items:

- no—rather no—neither yes nor no—rather yes—yes (questions 1, 2, 6)
- low—rather low—neither high nor low—rather high—very high (questions 3, 4, 5)

Moreover, the following questions could be answered in free text:

1. What did you particularly like about this teaching project?
2. What did you not like about this teaching project?
3. What would you improve in this teaching project?

### Methods

Quantitative values were presented as means and standard deviations, minimum, maximum, and quartiles. They were tested for normal distribution using the Shapiro–Wilk test. Since the distribution of age showed significant deviations from the normal distribution, a non-parametric Kruskal–Wallis test was used to analyze questions 1 to 6. Ordinally and nominally scaled values were displayed in absolute and percentage frequencies. Nominally scaled educational background and gender were compared with ordinally scaled answers to the questions in contingency tables, and were tested for association using the exact linear trend chi-square test. Bowker’s symmetry test was used to compare the answers to questions 3 and 4. The tests were two-sided, with a significance level of 5%. Alpha adjustment for multiple testing was not performed, and the results were interpreted accordingly. SPSS Statistics 25 (SPSS Inc. an IBM Company, Armonk, NY, USA) was used for statistical calculations.

## Results

### Description of the participating medical students

One-hundred and forty-three students attended the congress and filled in the questionnaire; 138 questionnaires were answered in full and used for further analysis. Ninety-eight students were female and their mean age was 23 years (range 19–36 years), Thirty-nine students were male and their mean age was 23 years (range 19–29 years). One student did not indicate his/her gender. While 64.1% of students had directly entered medical school, 35.9% had already completed a vocational training. Most of these students had worked as hospital or scrub nurses (16.2%) and emergency medical assistants (11.3%), whereas the others had been involved in medical, laboratory, biological, or pharmaceutical activities (8.4%).

### Analysis of questionnaires

**Fig. 1:**
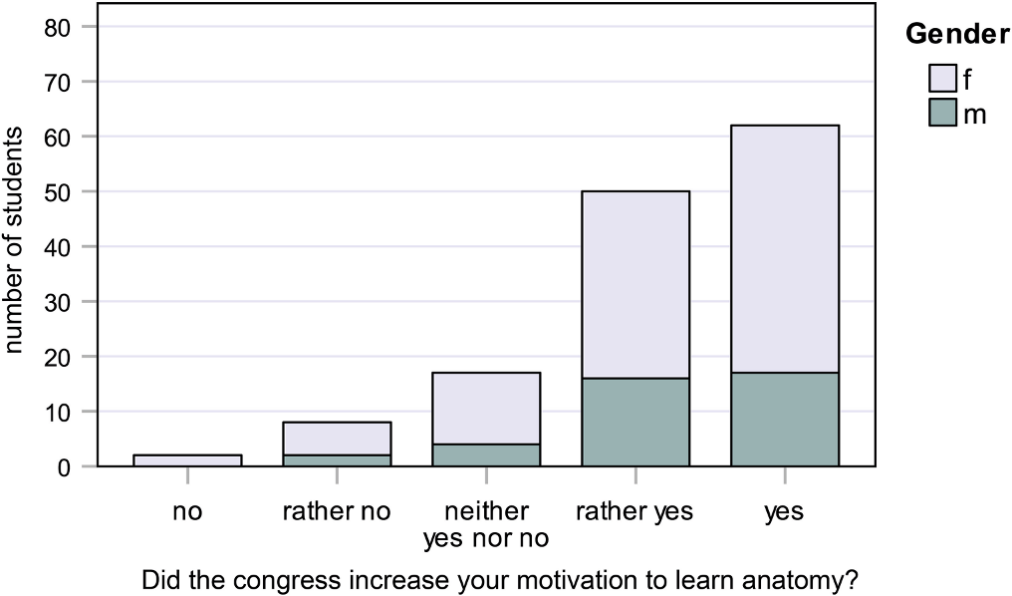
Responses to question 1: Did the congress increase your motivation to learn anatomy?

**Fig. 2:**
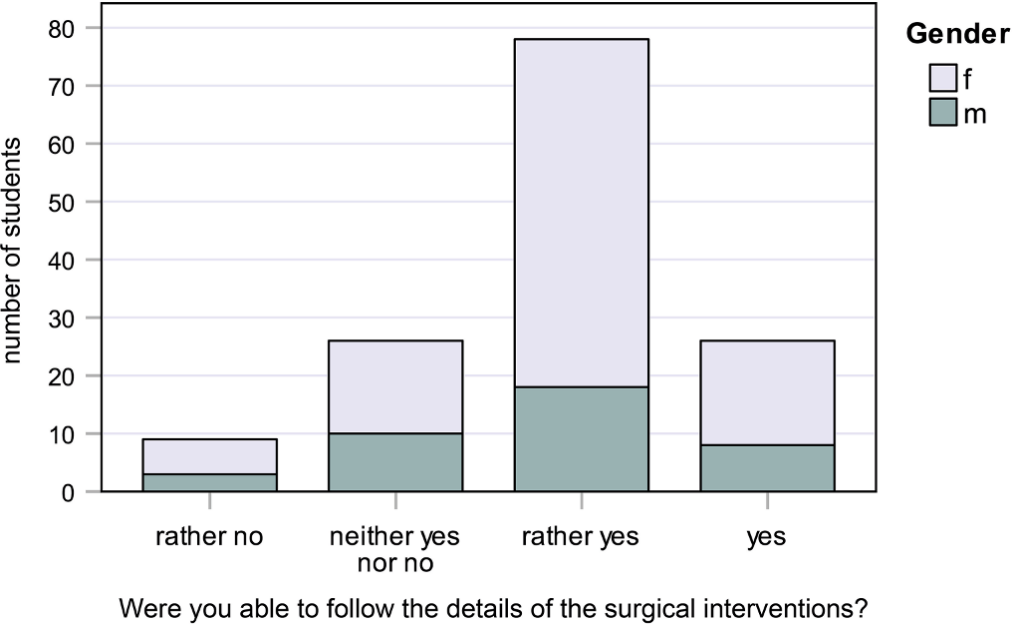
Responses to question 2: Were you able to follow the details of the surgical interventions?

**Fig. 3:**
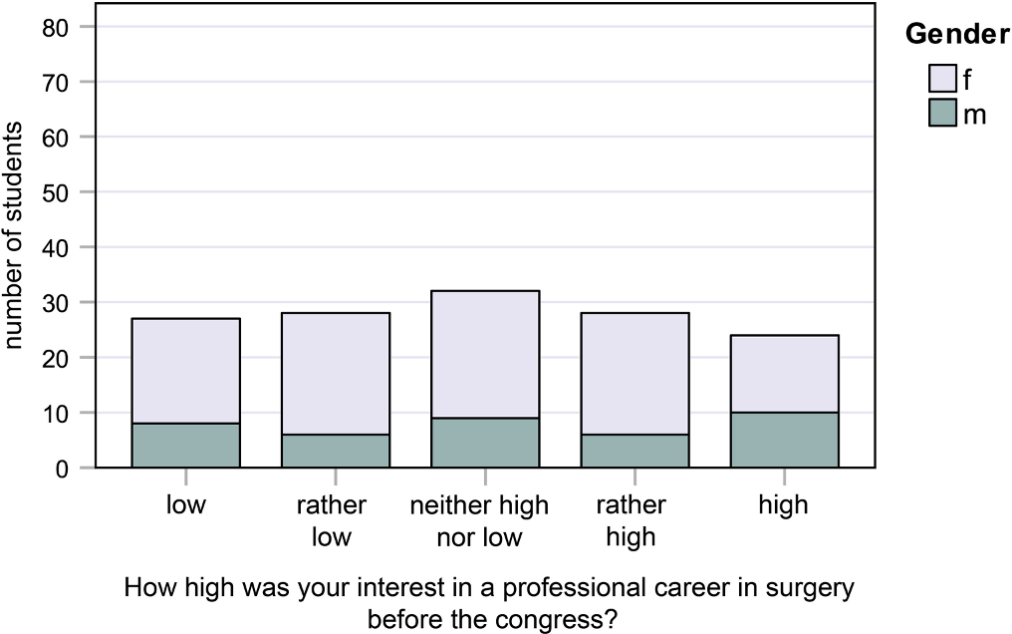
Responses to question 3: How high was your interest in a professional career in surgery *before* the congress?

**Fig. 4:**
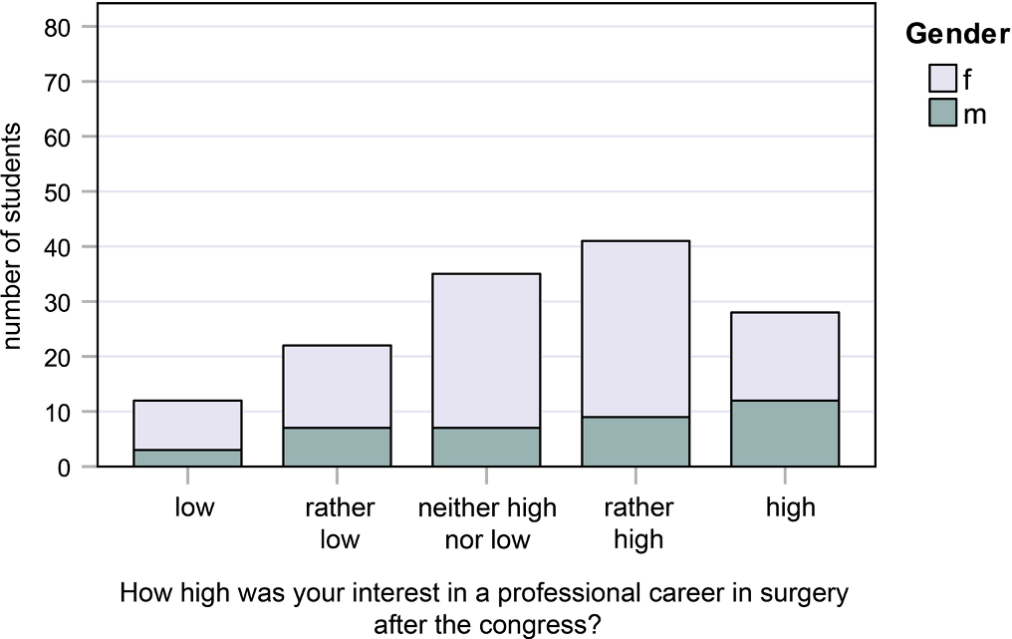
Responses to question 4: How high was your interest in a professional career in surgery *after* the congress?

**Fig. 5:**
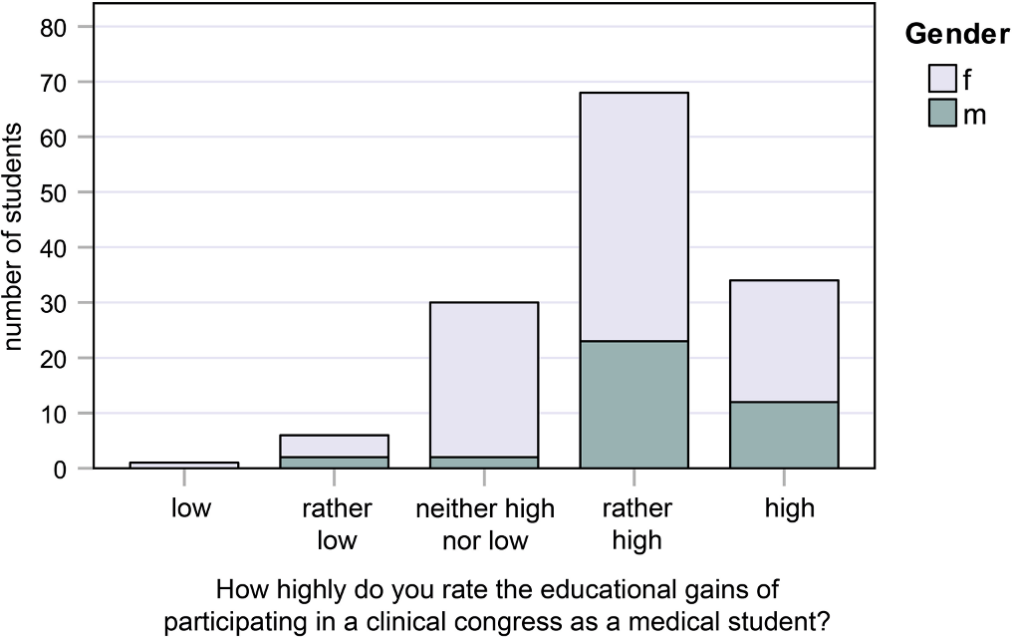
Responses to question 5: How highly do you rate the educational gains of participating in a clinical congress as a medical student?

**Fig. 6:**
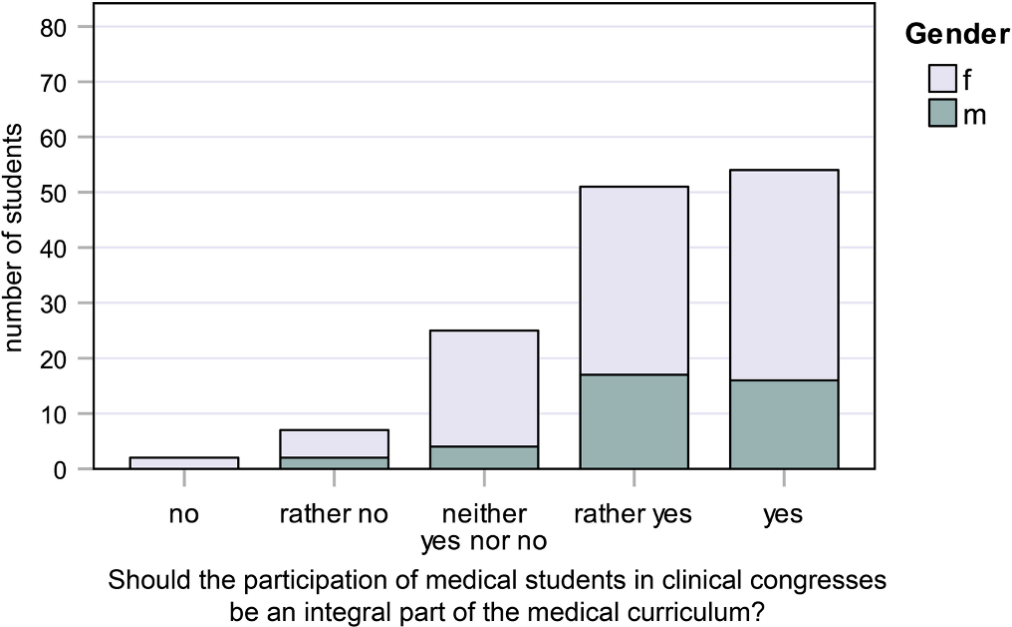
Responses to question 6: Should the participation of medical students in clinical congresses be an integral part of the medical curriculum?

Answers to the six questions (five-point Likert scale) were analyzed separately for male and female students, and are shown in bar charts (Figs. 1, 2, 3, 4, 5 and 6).

#### Question 1:

Did the congress increase your motivation to learn anatomy?

45.0% of female students and 43.6% of male students said “yes,” while 34.0% of female students and 41.0% of male students said “rather yes” (Fig. 1).

#### Question 2:

Were you able to follow the details of the individual surgical interventions?

18.0% of female students and 20.5% of male students said “yes,” while 60.0% of female students and 46.2% of male students said “rather yes.” None of the students said they were unable to follow the surgical interventions (Fig. 2).

#### Question 3:

How high was your interest in a professional career in surgery before the congress?

14.0% of female students and 25.6% of male students had had “very high” interest, while 19.0% of female students and 20.5% of male students had had “low” interest (Fig. 3).

#### Question 4:

How high was your interest in a professional career in surgery after the congress?

16.0% of female students and 31.6% of male students had “very high” interest, while 9.0% of female students and 7.9% of male students had “low” interest (Fig. 4).

#### Question 5:

How highly do you rate the educational gains of participating in a clinical congress as a medical student?

22.0% of female students and 30.8% of male students said “very high,” while 45.0% of female students and 59.0% of male students said “rather high” (Fig. 5).

#### Question 6:

Should the participation of medical students in clinical congresses be an integral part of the medical curriculum?

38.0% of female students and 41.0% of male students said “yes,” while 34.0% of female students and 43.6% of male students said “rather yes” (Fig. 6).

**Fig. 7:**
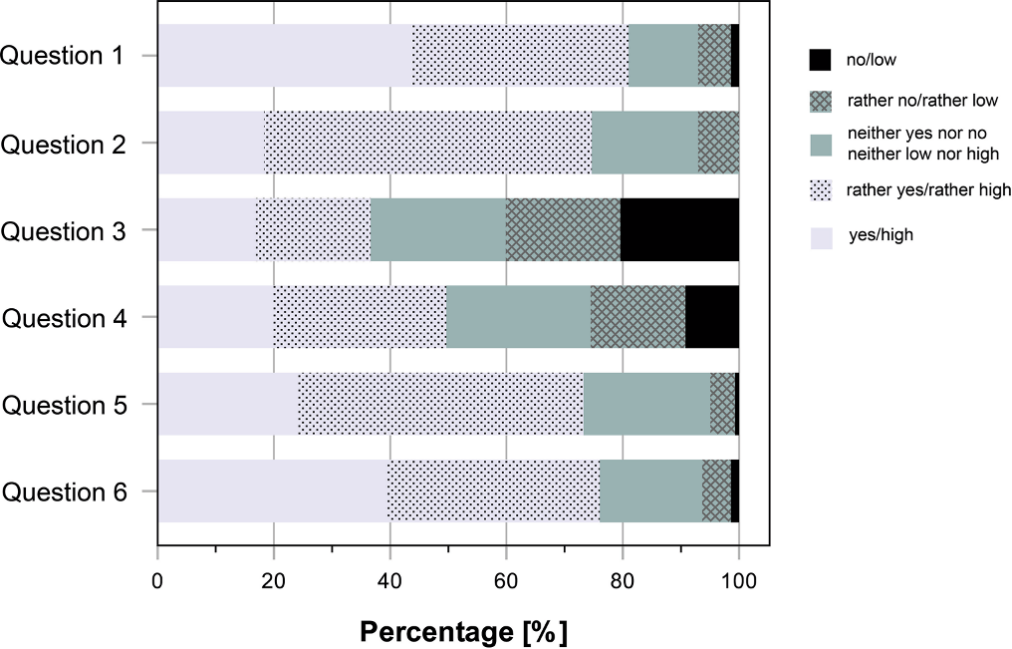
Summary of the answering behavior to questions 1–6

A summary of the answers to all questions is shown in Fig. 7.

### Influence of gender, age, and previous vocational training

The answers to all questions were analyzed with regard to the influence of gender, age, and previous vocational training. The influence of gender was only statistically significant (*p* = 0.041) for question 5 (How highly do you rate the educational gains of participating in a clinical congress as a medical student?). 89.8% of male students and 67.0% of female students rated the didactic gain very high and rather high, whereas 28.0% of female students and only 5.1% of male students said it was neither high nor low. No statistically significant difference was registered with regard to age (Kruskal–Wallis, *p* ≥ 0.05).

Comparison of the answers of students with and without previous vocational training revealed no statistically significant difference (chi-square test on linear trend, *p* ≥ 0.05). For a subgroup analysis, the students were divided into four groups: no vocational training (64.1%); hospital/scrub nurses (16.2%); emergency medical assistants (11.3%); medical, laboratory, biological, or pharmaceutical assistants (8.4%). Again, the answers were similar in all subgroups. Tests of significance could not be performed because of the small numbers in each group and the large number of answering options. Thus, no correlation was established between answering behavior and educational background.

### Interest in a professional career in surgery before and after the congress

**Fig. 8:**
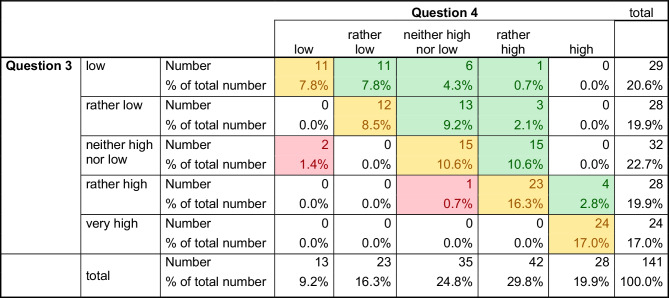
Interest in a professional career in surgery before and after the congress. Changes in response behavior concerning the interest in a professional career in surgery before (question 3) and after (question 4) the congress. The *green fields* indicate a change towards “high” (37.6%), the *yellow fields* indicate no change (60.3%), and the *red fields* indicate a change towards “low” (2.1%). The change in response behavior was significant (Bowker symmetry test, *p* < 0.001)

The students’ interest in a professional career in surgery was evaluated before and after the congress (questions 3 and 4) and analyzed by a cross table (Fig. 8). While 37.6% of students had greater interest, 60.3% reported no change, and 2.1% reported less interest than before. The change in responses between question 3 and 4 was significant (Bowker symmetry test, *p* < 0.001).

### Free-text evaluation of the teaching project

Finally, the students had the opportunity to evaluate the congress in free-text fields. They could express praise, criticism, and suggestions for improvement. The participants appreciated their active involvement in congress activities and the respect given to them by the surgical community. Their learning motivation and fascination for surgical disciplines were enhanced by the experience. On the other hand, owing to their limited knowledge, some attendees found it difficult to follow all surgical interventions and fully understand the diverse procedures. The students suggested a more detailed introduction to the clinical topics and surgical techniques presented at the congress, and adaptation of their own teaching modules to the contents of the congress. A summary of the most common free-text comments is given in Table 1.

**Table 1:** Free-text comments

What did you particularly like about this teaching project?	What did you not like about the teaching project?	What would you improve in this teaching project?
*Most common notions:* – Respect, appreciation, and the involvement of students (31.5%) – Motivation for enhanced learning activity due to practical relevance (19.6%) – No comment (31.5%)	*Most common notions:* – Lack of understanding of the content or subject (7.0%) – No comment (58.0%)	*Most common notions:* – Adaptation to the current teaching curriculum (21.7%) – Preparatory module for the contents of the congress in advance (14.0%) – No comment (51.7%)
*Representative quotations:* – “Doctors took time to explain anatomical structures explicitly to the students.” – “A lot of motivation to learn anatomy.” – “I got insights into areas that were not addressed during my preclinical studies.” – “More fascination for surgery.”	*Representative quotations:* – “The idea of students participating in the congress is good, but currently we are not involved in the topics discussed.” – “Too many changes of surgical interventions and topographic regions.” – “I could not follow so well because of my limited knowledge.”	*Representative quotations:* – “The topics of the congress should fit with the teaching curriculum for easier consolidation of what has been learned.” – “The spectrum of surgical techniques shown at the congress should be explained in advance.” – “More time for preparation.”

## Discussion

For the first time, medical students of the second academic year at the end of their preclinical period were given the opportunity to participate in a surgical congress. The congress was the 11th symposium of the German Society for Robot-Assisted Urology (*Deutsche Gesellschaft für Roboter-assistierte Urologie e.* *V.*, DRUS), and simultaneously the first interdisciplinary symposium for robot-assisted and digital surgery in urology, gynecology, and visceral surgery in Germany. As the surgical regions of interest presented at the congress fitted well with the anatomy lectures and dissection courses in macroscopic anatomy, the students were expected to gain valuable information about the importance of applied anatomy [8] for their clinical study period, and also about current technical innovations in minimally invasive surgery.

If the participation of medical students in a surgical congress is to be included in the curriculum as a matter of principle, one would first have to think about the funding. In our example, participation was free of charge. One consideration would be to involve the students financially to a small extent in the participation in the congress. In addition, the medical faculty, but also the participating clinics that host the congress, could share the costs. Another consideration would be to include the sponsors of the congress in the participation costs.

Although the study has considered many issues, there are some limitations worth mentioning: It would be interesting to investigate which medical specialty the students actually choose after graduation. Has it really become a surgical subject, as perhaps indicated on the questionnaire? For such an analysis, however, much more time would have to have passed, so that even then the congress would lose its topicality.

Another interesting starting point would be a modified study design in the sense of a case–control study. For example, one could investigate whether the medical specialty of congress participants differs from that of those who did not participate in a surgical congress.

Due to the strict separation of the preclinical and clinical study sections, preclinical medical students are largely unaware of the practical relevance of their theoretical knowledge. Although learning anatomy by means of lectures, textbooks, and dissection courses provides a basic understanding of the subject, these approaches are far removed from the clinical context and clinical applications. This didactic gap is particularly true for surgical disciplines and may result in a general lack of learning motivation as well as a specific lack of interest in pursuing a career in surgery.

### Interest in a career in surgery

Confronted with a declining number of applicants for general surgery programs, a study group in Texas [4] analyzed the factors that influence the career choice of medical students. One-hundred and eleven medical students in their fourth year of medical school participated in the study and ranked 18 items on a scale from 1 (not important) to 8 (very important). The factors were: career opportunities, academic opportunities, experience in core rotation/sub-internship, role model(s) in that specialty (mentors), length of training required, lifestyle during residency, work hours during residency, ability to obtain a residency position, concern about loans/debts, call schedule, lifestyle after training, work hours after training, financial rewards after training, intellectual challenge, patient relationships/interaction, prestige, future patient demographics, and gender distribution in the specialty. Only 17.1% students were interested in pursuing a surgical career because of career opportunities (*p* < 0.04) and prestige (*p* < 0.003). In contrast, lifestyle during residency (*p* < 0.0007), work hours during residency (*p* < 0.008), and the quality of the patient/physician relationship (*p* < 0.05) were all significantly negatively correlated with the choice of a career in surgery [4].

Schmidt et al. [9] analyzed six electronic databases concerning the current published literature about US medical students’ experience in surgery and the factors influencing their intention to pursue surgery as a career. The authors concluded that early introduction to surgery as well as recruitment strategies during the preclinical and clinical years of medical school are liable to substantially enhance the students’ interest in a surgical career [9].

These observations are in conformity with the results of our teaching project. Early introduction to surgical procedures during the preclinical period of medical school in the form of active participation in a congress culminated in greater interest in a subsequent surgical career. Comparison of the response behavior before and after the congress revealed that significantly more students would opt for a career in surgery after having attended the congress (*p* < 0.001).

### Enhanced learning motivation

The congress was focused on specific anatomical regions (pelvis, retroperitoneum), which were addressed by visceral surgeons, urologists, and gynecologists via video sessions and live surgeries. This interdisciplinary platform provided optimal first-hand impressions of clinically relevant aspects of anatomy, thus bridging the gap between theoretical knowledge and practical application. Subsequently, the students were more motivated to learn anatomy: 45.0% of female students and 43.6% of male students said “yes” to this question; 34.0% of female students and 41% of male students said “rather yes.”

In addition to live surgeries, medical students were invited to become familiar with virtual training devices and surgical simulators, which were presented during the congress to demonstrate and practice surgical processes and management [10–12]. The rationale for an early confrontation with these training tools was the observation that students using these devices in their medical curriculum were highly motivated, grasped the procedures rapidly, and became exceptionally skilled in the practical use of minimally invasive techniques [13].

### Role of gender in opting for a career in surgery

The percentage of female medical students has increased significantly throughout the world over the past decade [14]. This demographic shift is expected to considerably change not only the proportion of male versus female physicians, but also their respective medical careers of choice. A large-scale study from the Royal College of Surgeons in Ireland (RCSI) [15] analyzed the differences between male and female students’ perceptions of a surgical career. The results of the questionnaire (464 students, 40% males vs. 60% females) indicated that male students were significantly more influenced by remuneration than females (*p* < 0.001). In contrast, female students were significantly more influenced by part-time work (*p* < 0.001), parental leave (*p* < 0.001), working hours (*p* < 0.001), and length of residency (*p* = 0.003). The authors noted that the preference for a career in surgery declines with advancing academic years in medical school among male as well as female students. Medical students reported intense feelings of intimidation or being ignored during their surgical internships. Consequently, their enthusiasm for surgical disciplines declines consistently during their exposure to surgical practice. These findings, along with the importance of role modeling, underline the urgent need to address those factors that make surgery less appealing, especially to female medical graduates [15].

Chiu et al. [14] analyzed another interesting aspect of gender-specific differences: putative gender differences of medical students related to the acquisition of robotic suturing skills were examined. The students underwent a two-step DaVinci training; their performance was evaluated and compared. Female medical students performed significantly better in the virtual reality task of using a skin suturing pad and were able to complete more sutures with the robotic system than male students. However, no gender difference was noted with regard to the quality of the robotic suture [14].

The gender proportion in our study (98 females vs. 39 males, 2.5:1) confirms the general observation that the number of female medical students is two- to threefold higher than male medical students. However, when the results of the questionnaire were compared with regard to gender, a significant difference (*p* = 0.041) was only registered for question 5 (“How highly do you rate the educational gains of participating in a clinical congress as a medical student?”). While 22% of female students and 30.8% of male students said “very high,” 45% of female students and 59% of male students said “rather high,” suggesting that male students were more convinced about the educational benefit of the congress.

### Role of the generation shift in opting for a career in surgery

The steadily declining interest in pursuing a career in surgery appears to be related to the generation shift in favor of the currently dominant generations Y and Z, whose major focus is not surgery. Social sciences have divided the five living generations into the “builders,” “baby boomers,” and the respective generations “X, Y, and Z” [16]. Generation Y consists of persons born between 1980 and 1995 [17]. According to the data of the Federal Statistical Office published in 2015, about 22% of the total population and 20% of the workforce in Germany were born between 1980 and 1999 [18]. Generation Y is believed to be predominantly well educated, and their relatives frequently have a university degree. They prefer to work in teams than in strong hierarchies and consider the joy of work more important than status or prestige. More freedom of choice, the opportunity of self-fulfillment, and more time for family and leisure are the prime demands of generation Y [19]; they do not wish to subordinate everything else in their lives to their professional career, but strive for a work–life balance and a meaningful job [20]. These attitudes are in stark contrast to the circumstances in most surgical disciplines. The workload in surgical disciplines is far in excess of those envisioned by generation Y.

Lafraira et al. [21] performed a study to evaluate current attitudes, experiences, and expectations of residents belonging to the generation Y in surgical fields. One half of the residents were satisfied with the residency program. However, the proportion of dissatisfied reached 40% with regard to the number of surgical interventions, and 80% with regard to professional mentorship. Thus, 62% of the residents were not confident about performing operations after their residency. The authors conclude that current residents are less satisfied with their job and more critical of the quality of training and teaching. These altered attitudes match the profile of generation Y, which is more iconoclastic than previous generations [21].

A study from Germany [3] included 1098 medical students in a survey to gain insights into the mechanisms underlying the decision in favor of or against a career in surgery. The majority of students were of the opinion that surgery is an interesting and meaningful profession. However, the majority of the students (89% females, 81% males) were unwilling to choose a surgical specialty. While the students are certainly willing to spend a large amount of time on their professional lives, they demand the option of being able to plan their lives and achieve a satisfactory work–life balance. Flexible working hours and an existing childcare program were identified as predominant factors for all students, especially females. Prestige and salary were less relevant than “self-fulfillment” in terms of respectful interaction and a well-balanced professional and private life [3].

The present study did not focus in detail on the pros and cons raised by medical students concerning a career in surgery. However, the data retrieved from the questionnaire confirmed that medical students belonging to generation Y are not primarily interested in a time-consuming surgical career. While 37.4% and 39.5% of medical students had low/rather low interest, respectively, 23% were still undecided. However, active involvement in a surgically oriented congress obviously increased the students’ overall interest in opting for a professional career in surgical disciplines, including those belonging to generation Y.

## Conclusion

Apparently, learning motivation as well as the interest in a surgical career among medical students can be positively influenced during the early phase of their medical studies. Participation in a surgery congress had a twofold effect. On the one hand, the missing connection between the preclinical and clinical study period could be bridged by highlighting the practical clinical applications of the theoretical knowledge acquired by the students during their preclinical study period. On the other hand, the congress provided early insights into the tasks, challenges, and professional gains of a surgical profession, and thus established a realistic foundation for subsequent decisions concerning a medical subspecialty. The congress spanned a number of surgical disciplines, a large spectrum of live surgeries, and interactive training devices; this was considered especially suitable to provide large-scale multidimensional impressions of modern surgical practice [13].

It may be assumed that the stimulating experience of being directly involved in a surgical congress may not only increase the students’ learning motivation during medical school, but also their motivation to consider a career in surgery. At best, the enthusiasm induced by direct exposure to the fascinating aspects of surgical work is able to change the attitude of the current generation Y towards their later professional life and their willingness to face the required effort and tasks.

Moreover, medical faculties could be encouraged by the experiences gained in this pilot teaching project to incorporate the participation of medical students in medical congresses as an integral part of the curriculum. In particular, surgical societies should provide more options for medical students to take part in symposia, training workshops, and congresses. This would provide young academics with valuable insights into the spectrum of technical innovations and subspecialties. The motivation of the current and next generation to pursue a career in surgery against all odds will be achieved best by offering convincing reasons.
